# Associations between physical activity and sociodemographic and migration-related correlates among adults with a history of migration in Germany: results of a nationwide cross-sectional survey

**DOI:** 10.1186/s12889-026-28294-0

**Published:** 2026-06-30

**Authors:** Hanwei Zhang, Carmen Koschollek, Claudia Hövener, Julika Loss, Kristin Manz

**Affiliations:** 1 Section Sociology of Sport, Institute of Sociology and Gender Studies, German Sport University Cologne, Cologne, Germany; 2 Department for Epidemiology and Health Monitoring, Robert Koch Institute, Berlin, Germany; 3 Alice Salomon University of Applied Sciences Berlin, Berlin, Germany

**Keywords:** Physical activity, Movement behaviour, Cycling, Sports, Health inequalities, Migration

## Abstract

**Background:**

Promoting physical activity among the general population as well as among people with a history of migration is a crucial aspect of health promotion. However, we still lack a general understanding of physical activity levels and their potential correlates among adults with a history of migration living in Germany.

**Methods:**

The GEDA Fokus study, conducted by the Robert Koch Institute from 11/2021 to 05/2022, is a nationwide cross-sectional health interview survey of adults (18–79 years) with Croatian, Italian, Polish, Syrian or Turkish citizenship living throughout Germany (*n* = 6,038). The Aims of this study were (1) to assess the physical activity levels of adults with selected citizenships in Germany, specifically, adherence to World Health Organization (WHO) recommendations for aerobic activity and muscle strengthening, and in addition, cycling as a means of transport and (2) to identify sociodemographic, migration-related and psychosocial correlates of their physical activity behaviour.

**Results:**

Approximately 70% of the participants reported not achieving the WHO recommendations for aerobic physical activity and 75% did not achieve the recommendation for muscle strengthening. The physical activity levels among the participants were associated with a number of sociodemographic, migration-related and psychosocial factors. Multivariable regression analyses showed that men compared to women, adults with higher educational levels, higher subjective social status, better German language proficiency and a higher level of social support were more likely to achieve the WHO recommendations for physical activity. In addition, men compared to women and adults with higher educational levels were more likely to cycle as a means of transport.

**Conclusions:**

Overall, the study found that physical activity levels among adults of Croatian, Italian, Polish, Syrian or Turkish citizenship living in Germany were low in comparison to the general population, and there were significant associations between their physical activity behaviour and a range of sociodemographic, migration-related and psychosocial factors. Further in-depth research is needed, both from a public health and health sociology perspective, to identify barriers to physical activity among people with a history of migration and to derive appropriate measures for intervention planning.

## Background

The substantial health benefits of physical activity are widely recognised, including benefits in preventing chronic diseases as well as cancer and reducing the risk of death [1]. In Germany, a significant proportion of the population is still physically inactive. According to the GEDA 2019/2020-EHIS study, 48.0% of the population meets the WHO recommendations for aerobic physical activity (51.2% of men and 44.8% of women) [2]. While this level is considerably higher than the European Union (EU) average of 31.7%, Germany still lags behind top-performing European countries such as the Netherlands (60.8%), Sweden (55.5%), and Denmark (54.9%) [3].

Previous studies have shown that the physical activity levels of people living in Germany vary significantly according to different socio-demographic factors (e.g., age, gender and educational level). For example, older people, women and those with lower educational levels are generally less active [2]. In addition, migration has been found to be associated with lower levels of physical activity, particularly among women [4]. International reviews have shown similar socio-demographic corelates and have also identified migrant and ethnic minority populations in western countries as being at higher risk for physical inactivity [5, 6].

Although there is a growing body of research on the physical activity levels of the general population, there are still significant gaps in knowledge about the physical activity behaviour and associated factors of people with a history of migration living in Germany. People with a history of migration are an important part of the demographic structure in Germany, as 24.3% of the population living in Germany had an own or parental history of migration in 2022 [7]. However, it is not well understood which migration-related factors are associated with physical activity. Qualitative studies among specific migrant groups have identified various barriers, such as language difficulties, or time constraints, which may also change with the length of time spent in the new country [8, 9]. There is a lack of representative studies that help analyse correlates of physical activity in migrant populations, especially in Germany.

Therefore, we used data from the nationwide GEDA Fokus study, which surveyed adults with Croatian, Italian, Polish, Turkish, or Syrian citizenship living in Germany. The objectives of this study are (1) to describe the prevalence of physical activity behaviour in this sample in terms of aerobic physical activity, muscle-strengthening and specifically, cycling as a means of transport, and (2) to identify the socio-demographic, migration-related and psychosocial correlates of this physical activity behaviour [10].

## Methods

### Study design

For the current analyses, data of the health interview survey “Gesundheit in Deutschland aktuell: Fokus” (GEDA Fokus, German Health Update: Fokus) were used.

GEDA Fokus is a multilingual, cross-sectional study with a sequential mixed-mode design, conducted all over Germany from November 2021 until May 2022. A total of 120 primary sampling units in 99 cities and municipalities were randomly selected from all cities and municipalities all over Germany based on, inter alia, the proportion of the population without German citizenship in the administrative districts. In the second stage, adults aged 18–79 years were randomly selected from the population registers of the local registration offices based on their citizenship (first, second, or third, including dual citizens) as Croatian, Italian, Polish, Syrian, or Turkish. The selection of the citizenship groups followed model calculations described elsewhere [11]. Self-administered online and paper-based questionnaires were sequentially offered during the data collection phase. In larger cities, personal interviews were conducted by partly bilingual interviewers, either face to face in participants’ homes or by telephone. Questionnaires were available in Arabic, Croatian, Italian, German, Polish, and Turkish. Overall, 6,038 people took part, corresponding to a response rate of 18.4% (response rate 1 according to American Association for Public Opinion Research (AAPOR) [12]. The study design as well as the study population stratified by citizenship is described in more detail elsewhere [11].

### Dependent variables

The physical activity behaviour was assessed using the European Health Interview Survey - Physical Activity Questionnaire (EHIS-PAQ) [13]. Further information on the reliability and validity of the EHIS-PAQ can be found elsewhere [14]. Three indicators of physical activity were selected as the dependent variables: (1) achieving the WHO recommendations for aerobic physical activity, (2) muscle strengthening [15], and in addition (3) regular cycling as a means of transport (Table 1). In our analyses, we focused on cycling rather than walking, as cycling is typically performed at a higher intensity compared to walking and tends to have greater health benefits than walking, for example in terms of preventing cardiovascular diseases and obesity [16–20]. Therefore, cycling was included as a critical supplementary indicator of health-enhancing physical activity in our study. There is a lack of evidence providing information about a concrete threshold for the health benefits of cycling as a means of transport. Therefore, based on the knowledge that also low-to-moderate volumes of cycling can also have health benefits, we chose a threshold of “at least once a week” [21, 22].

### Independent variables

The following independent variables were selected: (a) sociodemographic variables: sex, age group, educational level and subjective social status; (b) migration-related variables: duration of residence, and self-assessed German language proficiency; and (c) psychosocial variables: self-reported social support and sense of belonging to the society in Germany. A detailed overview of the dependent and independent variables is presented in Table 1.

**Table 1:** Definition of variables

Dependent variable: Physical Activity	Categorization
• Achieving the WHO recommendations for aerobic physical activity Based on the sum of physical activity during leisure time and cycling as a means of transport [14]	< 150 min/ Week, ≥ 150 min/ Week
• Achieving the WHO recommendations for muscle strengthening Based on number of times reported by respondents [14]	< Twice/ Week ≥Twice/ Week
• Cycling regularly as a means of transport Based on number of times reported by respondents [14]	< Once/ Week ≥Once/ Week
Independent variables:	
Socio-demographic variables	
• Sex: Based on biological sex assigned at birth	Men Women
• Age group: Age categorised into three groups	18–39 years 40–59 years 60–79 years
• Educational level: Based on the ISCED-2011 [23] classification standard	Low (ISCED 1–2) Medium (ISCED 3–4) High (ISCED 5–8)
• Subjective social status: Based on the MacArthur Scale of Subjective Social Status [24, 25]	Low (values 1–4) Medium (values 5–6) High (values 7–10)
Migration-related variables:	
• Duration of residence: Based on country of birth and year of moving to Germany for those born abroad	< 5 years ≥ 5 years (but less than a lifetime) Since birth
• German language proficiency Based on questions on native language and the self-assessed German language proficiency of those not indicating German as their native language	Native/ Very good Good/ Moderate Bad/ Very bad
Psychosocial variables:	
• Social support Based on the Oslo 3-item Social Support Scale [26]	Poor (values 3–8) Moderate (values 9–11) Strong (values 12–14)
• Sense of belonging to the society in Germany Based on the question ‘How much do you feel you belong to the society in Germany?” [27]	Very strongly/ Strongly Partly Barely/ Not at all

### Statistical analyses

Statistical analyses were performed using Stata version 17.0 survey procedures for complex samples. Analyses were performed using a weighting factor to ensure that the sample was representative of the population with corresponding citizenships. Results are presented as prevalences with corresponding 95% confidence intervals (95% CI). A statistically significant difference is assumed at a p-value less than 0.05.

Bivariate logistic regression analyses were performed to determine the associations between each physical activity indicator and the socio-demographic, migration-related and psychosocial variables, yielding unadjusted odds ratios (ORs) with corresponding 95% confidence intervals (CIs). Subsequently, multivariable adjusted odds ratios (OR) with corresponding 95% CIs were calculated using logistic regression analyses. The analyses were run separately for each of the three dependent variables (aerobic activity, muscle strengthening, and cycling) and included the socio-demographic (sex, age group, educational level, subjective social status), the migration-related (duration of residence, German language proficiency) and the psychosocial (social support, sense of belonging to the society in Germany) independent variables. All multivariable analyses were additionally adjusted for citizenship (Croatian, Italian, Polish, Syrian and Turkish) according to residents’ registration offices to account for different response behaviour between citizenship groups [28].

## Results

### Study sample

A total of 6,038 participants took part in the survey, consisting of 53.8% men and 46.2% women (% weighted). The participants had a mean age of 41.6 years. Table 2 shows the descriptives of the sociodemographic, migration-related and psychosocial characteristics of the participants.

**Table 2:** Description of the study sample according to sociodemographic, migration-related and psychosocial variables

Variable:	Categorization	*n*	% [95%CI]
Sociodemographic characteristics:			
Sex	Men Women	3055 2983	53.8 [51.0- 56.5] 46.2 [43.5–49.0]
Age group	18–39 years 40–59 years 60–79 years	3024 2156 858	46.5 [43.7–49.4] 37.9 [35.9–39.9] 15.6 [13.4–18.0]
Educational level	Low Medium High *Missing*	1704 2260 2042 32	45.3 [42.4–48.3] 39.9 [37.7–42.2] 14.2 [12.5–16.1] 0.5 [0.3–0.9]
Subjective social status	Low Medium High *Missing*	1920 2266 1597 255	31.7 [28.9–34.6] 39.6 [36.8–42.5] 24.1 [22.0- 26.4] 4.5 [3.5–5.9]
Migration-related characteristics:			
Duration of residence	< 5 years ≥ 5 years Since birth *Missing*	2255 2507 1189 87	22.7 [19.3–26.5] 56.2 [53.0- 59.5] 19.5 [17.3–21.9] 1.5 [0.9–2.5]
German language proficiency	Native/ Very good Good/ Moderate Bad/ Very bad *Missing*	2570 2839 538 91	44.4 [42.3–46.6] 46.5 [44.4–48.6] 7.2 [6.2–8.2] 1.9 [1.4–2.6]
Psychosocial characteristics:			
Social support	Poor Moderate Strong *Missing*	1561 3062 1356 59	25.7 [23.0- 28.5] 49.1 [46.6–51.5] 24.3 [21.5–27.2] 1.0 [0.6–1.5]
Sense of belonging	Very strongly/ Strongly Partly Barely/ Not at all *Missing*	3655 1824 548 11	63.0 [60.6–65.4] 28.7 [26.9–30.6] 7.9 [6.8–9.1] 0.3 [0.1–0.8]
Outcome variables:			
Aerobic physical activity recommendation achieved	Yes No *Missing*	1941 4032 65	28.5 [26.4–30.7] 70.0 [67.7–72.2] 1.5 [1.0- 2.2]
Muscle strengthening recommendation achieved	Yes No *Missing*	1551 4391 96	22.6 [20.5–24.8] 75.0 [72.5–77.4] 2.4 [1.7–3.3]
Cycling for travel at least once a week	Yes No *Missing*	1532 4392 114	22.0 [19.5–24.6] 75.4 [72.7–77.8] 2.7 [2.1–3.4]

*n* Unweighted, *%* Weighted, *CI* Confidence interval

### Physical activity levels

Of the participants, 28.5% (95% CI: 26.4–30.7) achieved the recommended 150 min or more of aerobic physical activity per week, and 22.6% (95% CI: 20.5–24.8) met the recommended level of at least two muscle-strengthening sessions per week. Of the participants, 22.0% (95% CI: 19.5–24.6) cycled at least once a week as a means of transport.

#### Bivariate logistic regression analyses

Bivariate logistic regression analyses showed that all selected variables were found to be significantly associated with the achievement of WHO recommendation for aerobic physical activity (Table 3).

**Table 3:** Results of the bivariate and multivariable logistic regression analyses

	Aerobic physical activity recommendation achieved				Muscle strengthening recommendation achieved				Cycling for travel at least once a week			
	Bivariate regression		Multivariable regression*		Bivariate regression		Multivariable regression*		Bivariate regression		Multivariable regression*	
	OR [95%CI]	*p*-value	OR [95%CI]	*p*-value	OR [95%CI]	*p*-value	OR [95%CI]	*p*-value	OR [95%CI]	*p*-value	OR [95%CI]	*p*-value
Sex												
Men	1.35 [1.11–1.65]	**0.003**	1.41 [1.12–1.77]	**0.004**	1.52 [1.24–1.86]	**< 0.001**	1.54 [1.22–1.93]	**< 0.001**	1.65 [1.31–2.06]	**< 0.001**	1.68 [1.35–2.10]	**< 0.001**
Women	1.00 (ref.)		1.00 (ref.)		1.00 (ref.)		1.00 (ref.)		1.00 (ref.)		1.00 (ref.)	
Age group												
18–39 years	1.69 [1.27–2.24]	**< 0.001**	1.48 [1.02–2.15]	**0.041**	2.07 [1.61–2.67]	**< 0.001**	1.98 [1.33–2.94]	**0.001**	1.25 [0.92–1.71]	0.152	1.07 [0.74–1.55]	0.719
40–59 years	1.16 [0.84–1.60]	0.378	1.12 [0.78–1.61]	0.527	1.26 [0.93–1.69]	0.129	1.26 [0.88–1.81]	0.203	1.03 [0.79–1.36]	0.816	0.95 [0.70–1.28]	0.727
60–79 years	1.00 (ref.)		1.00 (ref.)		1.00 (ref.)		1.00 (ref.)		1.00 (ref.)		1.00 (ref.)	
Educational level												
Low	1.00 (ref.)		1.00 (ref.)		1.00 (ref.)		1.00 (ref.)		1.00 (ref.)		1.00 (ref.)	
Medium	2.37 [1.96–2.88]	**< 0.001**	1.74 [1.41–2.15]	**< 0.001**	1.69 [1.33–2.15]	**< 0.001**	1.33 [1.00-1.77]	0.050	1.81 [1.36–2.41]	**< 0.001**	1.64 [1.24–2.16]	**0.001**
High	2.83 [2.19–3.65]	**< 0.001**	2.11 [1.59–2.79]	**< 0.001**	2.07 [1.59–2.70]	**< 0.001**	1.58 [1.16–2.14]	**0.004**	2.00 [1.48–2.71]	**< 0.001**	1.76 [1.29–2.38]	**< 0.001**
Subjective social status												
Low	1.00 (ref.)		1.00 (ref.)		1.00 (ref.)		1.00 (ref.)		1.00 (ref.)		1.00 (ref.)	
Medium	1.48 [1.20–1.82]	**< 0.001**	1.24 [1.00-1.55]	0.055	1.49 [1.15–1.94]	**0.003**	1.43 [1.11–1.86]	**0.007**	1.13 [0.85–1.50]	0.411	1.04 [0.76–1.41]	0.822
High	1.59 [1.19–2.11]	**0.002**	1.31 [0.98–1.74]	0.064	1.67 [1.24–2.24]	**0.001**	1.57 [1.17–2.10]	**0.003**	1.30 [0.94–1.80]	0.106	1.14 [0.81–1.61]	0.439
Duration of residence												
< 5 years	0.59 [0.44–0.80]	**0.001**	0.97 [0.68–1.40]	0.885	0.72 [0.52–0.98]	**0.038**	0.81 [0.53–1.25]	0.335	1.27 [0.95–1.70]	0.105	1.55 [1.02–2.34]	**0.041**
≥ 5 years	0.60 [0.44–0.81]	**0.001**	0.84 [0.59–1.17]	0.297	0.63 [0.48–0.82]	**0.001**	0.92 [0.67–1.26]	0.600	1.03 [0.78–1.36]	0.840	1.13 [0.82–1.57]	0.454
Since birth	1.00 (ref.)		1.00 (ref.)		1.00 (ref.)		1.00 (ref.)		1.00 (ref.)		1.00 (ref.)	
German language proficiency												
Native/ very good	2.85 [1.82–4.48]	**< 0.001**	2.14 [1.32–3.46]	**0.002**	2.47 [1.55–3.94]	**< 0.001**	2.04 [1.20–3.48]	**0.009**	1.27 [0.80–2.03]	0.308	1.35 [0.87–2.08]	0.176
Good/ moderate	1.38 [0.89–2.14]	0.149	1.37 [0.88–2.14]	0.160	1.68 [1.03–2.74]	**0.036**	1.77 [1.10–2.86]	**0.020**	1.15 [0.71–1.85]	0.571	1.29 [0.82–2.02]	0.260
Bad/ very bad	1.00 (ref.)		1.00 (ref.)		1.00 (ref.)		1.00 (ref.)		1.00 (ref.)		1.00 (ref.)	
Social support												
Poor	1.00 (ref.)		1.00 (ref.)		1.00 (ref.)		1.00 (ref.)		1.00 (ref.)		1.00 (ref.)	
Moderate	1.46 [1.13–1.89]	**0.004**	1.31 [0.98–1.76]	0.070	1.63 [1.22–2.16]	**0.001**	1.82 [1.37–2.44]	**< 0.001**	1.20 [0.96–1.51]	0.115	1.16 [0.89–1.50]	0.275
Strong	1.69 [1.22–2.35]	**0.002**	1.46 [1.04–2.05]	**0.027**	1.91 [1.36–2.67]	**< 0.001**	2.09 [1.50–2.91]	**< 0.001**	1.12 [0.88–1.44]	0.354	1.11 [0.82–1.51]	0.508
Sense of belonging												
Very strongly/ strongly	1.62 [1.13–2.33]	**0.009**	1.24 [0.87–1.76]	0.225	1.35 [0.88–2.07]	0.173	0.80 [0.53–1.21]	0.282	1.42 [1.00-2.01]	0.049	1.36 [0.92–2.03]	0.123
Partly	1.31 [0.93–1.85]	0.117	1.20 [0.83–1.74]	0.337	1.33 [0.90–1.98]	0.156	0.99 [0.67–1.47]	0.963	1.29 [0.89–1.87]	0.180	1.27 [0.85–1.88]	0.242
Barely/ not at all	1.00 (ref.)		1.00 (ref.)		1.00 (ref.)		1.00 (ref.)		1.00 (ref.)		1.00 (ref.)	

*OR* Odds ratio, *CI* Confidence interval, *ref.* Reference category, Bold values indicate statistical significance (*p* < 0.05)

*Adjusted for citizenship according to residents’ registration offices

Except for the sense of belonging to the society in Germany, all selected variables were significantly associated with achieving the WHO recommendations for muscle strengthening as well.

Sex and educational level were found to be significantly associated with cycling behaviour in the bivariate logistic regression analyses.

#### Multivariable logistic regression analyses

Men compared to women had a 1.4 times higher chance of achieving the WHO recommendation for aerobic physical activity (Table 3). Younger adults (aged 18–39) had a 1.5 times higher chance than older adults (aged 60 to 79), and those with a medium or high educational level had a 1.7 or 2.1 times higher chance, respectively. In addition, the chance of meeting the aerobic physical activity recommendation was 2.1 times higher for those with native or very good compared to bad or very bad German proficiency and 1.5 times higher with a strong social support compared to poor social support.

A higher chance to achieve the recommendation for muscle-strengthening was observed among men compared to women (OR: 1.5), younger adults (aged 18–39) compared to older adults aged 60 to 79 years (OR: 2.0), and those with a high educational level (OR high vs. low: 1.6) (Table 3). A higher chance was additionally observed among those with a higher subjective social status (OR medium vs. low: 1.4; OR high vs. low: 1.6), better German language proficiency (OR native/very good vs. bad/very bad: 2.0; OR good/moderate vs. bad/very bad: 1.8), and stronger social support (OR moderate vs. poor: 1.8; OR strong vs. poor: 2.1).

A higher chance to cycle as a means of transport at least once a week was found for men compared to women (OR: 1.7), for people with a medium or high level of education compared to those with a low educational level (OR medium vs. low: 1.6; OR high vs. low: 1.8), and for people who have lived in Germany for less than five years compared to those who were born in Germany (OR: 1.6) (Table 3).

## Discussion

Our study showed that the overall level of physical activity among adults with a history of migration in Germany was comparatively low, with less than a third achieving the WHO recommendations for aerobic and muscle-strengthening physical activity. A higher chance of failing to meet the physical activity recommendations was observed among women, older people, those with a low educational level, a low subjective social status (only meeting the muscle strengthening recommendation), a low level of social support and a lower proficiency in the German language. Furthermore, women, those with lower levels of education and those who have lived in Germany for a longer time had a lower chance to regularly use cycling as a means of transport.

In our study, only 28.5% of participants met the WHO recommendation for aerobic activity, and 22.6% met the recommendation for muscle strengthening. These figures stand in stark contrast to the general population in Germany, as reported by the GEDA 2019/2020-EHIS study, where 48.0% (51.2% of men and 44.8% of women) met the recommended levels [2]. Similarly, the prevalence for muscle-strengthening in our sample is lower than the 36.3% reported for the general population in Germany [29]. This disparity highlights health inequalities, suggesting that people with a history of migration are far less active than the general population. However, this direct comparison warrants careful interpretation as the periods of data collection varied, which could have affected physical activity behaviour due to the pandemic. The GEDA 2019/2020-EHIS data were collected before and during the first months of the COVID-19 pandemic, whereas our study (GEDA Fokus) was conducted during the second part of the pandemic (2021–2022), a period known to have introduced additional barriers to physical activity. Second, the GEDA 2019/2020-EHIS survey was conducted via telephone interviews. This method may be more susceptible to social desirability bias, potentially leading to an overestimation of activity levels in the data. Finally, the GEDA 2019/2020-EHIS sample itself includes individuals with a history of migration as well, hence, we cannot directly compare between people with and without a history of migration.

### Correlates of physical activity among respondents

Men in the sample were more likely than women to achieve the physical activity recommendations. These gender differences in physical activity are consistent with previous research findings among migrants as well as in the general population living in Germany [30–34]. This disparity may be attributed to gender-specific socialization patterns, which tend to provide men with greater motivation and social support to engage in physical activity [35].

Age was found to be significantly associated with aerobic physical activity and muscle-strengthening, with younger adults (18–39 years) being more likely to be physically active, while the older group (60 years and older) was less active. This finding reflects an observation not only in Germany [2] but also across broader Europe [3], where physical activity levels consistently decline with increasing age. While biological factors such as decreasing health and fitness levels and frailty may contribute to lower levels of physical activity among older adults in the general population, other barriers may also be relevant for older adults with a history of migration, such as social isolation, discrimination, or low social participation [36].

We also found associations between higher levels of education and more frequent participation in physical activity, which is consistent with studies from the general population [2], but has also been observed in other research on migrant and ethnic minority groups [37, 38]. Individuals with higher levels of education are more likely to have better health literacy, and therefore may be more likely to understand the health benefits of physical activity and engage in such activities intentionally [39]. In contrast, those with lower levels of education are often more likely to be engaged in more physically demanding jobs [40], a factor which may also be particularly relevant for migrant populations in certain labor sectors [41]. This may further reduce their chances of engaging in health-enhancing physical activity during their leisure time.

Regarding subjective social status, our multivariable analyses reveal a nuanced pattern: while subjective social status remained a significant independent predictor for muscle strengthening, its association with aerobic physical activity was attenuated and lost statistical significance after adjusting for other variables. This suggests that for aerobic activity, the association with subjective status may be largely mediated by other socio-demographic factors. In contrast, the association with muscle strengthening implies a distinct link, which aligns with broader research indicating associations between subjective status and health behaviours in migrant populations [42]. This distinction may be due to the fact that muscle strengthening often relies more on specific resources; individuals with a higher subjective social status may have lower perceived stress levels and greater opportunities to engage in leisure-time muscle strengthening activity, such as joining training clubs or gyms [43].

In addition to socio-demographic correlates, this study identified other significant correlates of physical activity, particularly migration-related and psychosocial factors: those with better German language proficiency and higher levels of social support were more likely to meet the recommendations for physical activity. It is likely that there exists a complex interplay between these factors and physical activity. Improved language proficiency of the majority language (i.e. the language spoken by the majority population in a specific country or region) offers people with a history of migration better chances for social integration [44]. This integration, in turn, enables them to access health information, communicate effectively with healthcare providers, and actively participate in public physical activities based on their individual needs [45]. Language difficulties can also prevent individuals from obtaining information about sports and exercise programs [9]. Additionally, strong social support provides people, either with or without history of migration, with the necessary resources and decision-making assistance from their families and communities, fostering a supportive environment for sustained physical activity and long-term adherence [46]. In turn, it appears that engaging in physical activity provides distinctive psychosocial benefits. A recent study in Germany found that among middle-aged and older adults, migrants benefit to a larger extent from following physical activity recommendations in terms of reduced loneliness compared to the non-migrant population [47].

In this study, regular cycling as a means of transport was only found to be significantly associated with sex, education, and duration of residence in Germany in the multivariable regression analysis.

Regarding sex, an earlier survey investigating cycling among a group of adults with personal migration experience in Germany (IntegRADtion) also found higher rates of cycling among men than women [48]. This gender difference in cycling behaviour is also found in the general population living in Germany: a nationwide study in 2021 showed that 40% of men cycled at least once a week as a means of transport, but only 29% of women [49]. A lack of safe environments, cultural norms, and fear of harassment could explain women’s lower cycling rates [50]. In contrast, gender differences in cycling are significantly reduced in societies with safe cycling infrastructure and relatively gender-equal societies [50].

The lower prevalence of cycling as a means of transport among people with low levels of education compared to those with higher levels of education found in our study is also consistent with findings in the general population living in Germany [49, 51]. One explanation may lie in different attitudes towards cycling as a mode of transport. People with low educational levels may avoid cycling as a mode of transport because it may be associated with a low social status [51]. In contrast, people with higher educational level often see cycling as fashionable and ethical because of its environmental and fitness benefits [51].

Duration of residence was negatively associated with the chance of cycling as a means of transport. Newcomers may choose cycling as their primary mode of transport due to unfamiliarity with the new environment, such as public transport routes, but also because it offers a significantly cheaper alternative to public transport or car ownership, thereby addressing the potential financial pressure [8]. However, in contrast to our results, the findings of the IntegRADtion study by Schaefer & Francke indicate that adults living in Germany who grew up abroad were less likely to choose cycling as a mode of transport than the group who grew up in Germany [48]. In addition, the IntegRADtion study found that the main barriers and motivators for cycling among adults with migration experience were barely different from those of the general population. Therefore, the various interactions between the duration of immigrants’ residence and their characteristics still need to be further investigated in the future.

### Recommendations for policy and practice

Interventions to promote physical activity among adults with a history of migration should address several dimensions. First, they should consider the heterogeneity of different sub-populations with migration history [52]. This heterogeneity is characterized by multiple dimensions of socioeconomic status, and community resources, and therefore presents different challenges to their physical activity participation. Therefore, in order to provide needs-based support frameworks for physical activity, interventions should be tailored to the specific social, economic, and environmental needs of population groups. To establish successful approaches to promoting physical activity among people with a history of migration, future interventions should include a participatory approach, as recommended by the WHO [53]. This approach ensures that the target group is meaningfully involved in the planning, delivery, and evaluation of interventions, thereby enhancing their relevance and effectiveness.

In addition, research is needed to better understand the barriers to physical activity of people with a history of migration, with a focus on women, people with a low subjective social status and lower levels of German language proficiency. Furthermore, it should be investigated how sports facilities and public spaces can be adapted to meet the diverse needs of adults with a history of migration for physical activity [54]. Based on our finding that low proficiency in German language predicted significant physical inactivity, increasing the number of sports classes offered in different languages could increase the physical activity behaviour of people with low German language proficiency, in addition to appropriate ways of promoting these sports classes to the respective groups.

### Strengths

This study has several strengths: First, it is the first to use nationwide cross-sectional survey data to examine the association between physical activity and a wide range of correlates among adults with selected citizenships in Germany, including socio-demographic, migration-related and psychosocial factors. Second, the study design was complex and used different methods, including home visits, to recruit individuals with a history of migration. Finally, by filling this research gap, this study provides a valuable new source of data that serves as an important reference for the development of relevant policies and the selection of directions for future in-depth research.

### Limitations

A limitation of this study may lie in the selection of variables. Despite efforts to include various selection of variables, further potential correlates of the physical activity behaviour of the participants were not included in the questionnaire, such as physical literacy or environmental factors. Furthermore, occupational physical activity was not included in the statistical analysis due to its potential negative health effects [55] and walking, an important public health relevant behaviour, was not included in order to not avoid overwhelming the scope of the analyses. These aspects remain a potential area for future research.

In addition, the variables in this study were based on participants’ self-reports, which may have biased the results on their physical activity levels due to recall and social desirability bias. Related to measurement, although the questionnaire was available in six languages, we cannot exclude that comprehension difficulties due to varying language proficiency or nuances lost in translation may have led to measurement error. Another methodological limitation is the binary operationalisation of our dependent variables (i.e., meeting the recommendation or not). This approach, while clear, simplifies the complexity of physical activity.

Another issue that needs to be mentioned is that the generalizability of our findings is limited to people with the five selected citizenships, but not to all people with a history of migration living in Germany. Additionally, the response rate of 18.4% is comparatively low which probably introduced nonresponse bias. In line with that, regarding the sample composition, it appears that a large majority of participants reported at least good German language proficiency. Consequently, the subsamples for those with low German language proficiency and a low sense of belonging to the society were relatively small. This imbalance is reflected in the wider confidence intervals observed for these groups in the analyses, indicating lower statistical precision. Therefore, results concerning these specific subgroups should be interpreted with caution.

Finally, the cross-sectional design means that causal relationships cannot be established. Longitudinal studies are needed to clarify the causal pathways.

## Conclusions

Overall, the study found that physical activity levels among adults with Croatian, Italian, Polish, Syrian or Turkish citizenship in Germany were low in comparison with the general population, and there were significant associations between their physical activity behaviour and a range of socio-demographic, migration-related and psychosocial correlates.

Further in-depth research is necessary and valuable from both a public health and health sociology perspective. It is necessary for policy and intervention planning to pay particular attention to addressing existing social inequities in order to effectively promote physical activity among people with a history of migration for their health and well-being.

## Data Availability

The dataset supporting the conclusions of this article cannot be made publicly available because informed consent from study participants did not cover public deposition of data. However, the minimal dataset underlying the findings is archived in the Research Data Centre at the Robert Koch Institute and can be accessed by researchers on reasonable request. On-site access to the data set is possible at the Secure Data Centre of the Robert Koch Institute’s Research Data Centre. Requests should be submitted by e-mail to fdz@rki.de.

## Abbreviations

CI: Confidence Interval
GEDA: Gesundheit in Deutschland aktuell
ISCED: International Standard Classification of Education (ISCED)
OR: Odds Ratio
WHO: World Health Organization
